# Carbon-coated Fe_3_O_4_ core–shell super-paramagnetic nanoparticle-based ferrofluid for heat transfer applications†

**DOI:** 10.1039/d1na00061f

**Published:** 2021-02-08

**Authors:** Mohd Imran, Nasser Zouli, Tansir Ahamad, Saad M. Alshehri, Mohammed Rehaan Chandan, Shahir Hussain, Abdul Aziz, Mushtaq Ahmad Dar, Afzal Khan

**Affiliations:** Department of Chemical Engineering, Faculty of Engineering, Jazan University P.O. Box. 706 Jazan 45142 Saudi Arabia; Department of Chemistry, College of Science, King Saud University P.O. Box 2455 Riyadh 11451 Saudi Arabia; Colloids and Polymers Research Group, School of Chemical Engineering, Vellore Institute of Technology Vellore Tamilnadu 632014 India chandan1816@gmail.com +91-04162202668; Department of Electrical Engineering, Faculty of Engineering, Jazan University P.O. Box. 706 Jazan 45142 Saudi Arabia; Department of Mechanical Engineering, Faculty of Engineering, Jazan University P.O. Box. 706 Jazan 45142 Saudi Arabia; Center of Excellence for Research in Engineering Materials, College of Engineering, King Saud University Riyadh 11421 Kingdom of Saudi Arabia; State Key Laboratory of Silicon Materials, School of Materials Science and Engineering, Zhejiang University Hangzhou 310027 China afzalkhan@zju.edu.cn +86-15669097732

## Abstract

Herein, we report the investigation of the electrical and thermal conductivity of Fe_3_O_4_ and Fe_3_O_4_@carbon (Fe_3_O_4_@C) core–shell nanoparticle (NP)-based ferrofluids. Different sized Fe_3_O_4_ NPs were synthesized *via* a chemical co-precipitation method followed by carbon coating as a shell over the Fe_3_O_4_ NPs *via* the hydrothermal technique. The average particle size of Fe_3_O_4_ NPs and Fe_3_O_4_@C core–shell NPs was found to be in the range of ∼5–25 nm and ∼7–28 nm, respectively. The thickness of the carbon shell over the Fe_3_O_4_ NPs was found to be in the range of ∼1–3 nm. The magnetic characterization revealed that the as-synthesized small average-sized Fe_3_O_4_ NPs (*ca.* 5 nm) and Fe_3_O_4_@C core–shell NPs (*ca.* 7 nm) were superparamagnetic in nature. The electrical and thermal conductivities of Fe_3_O_4_ NPs and Fe_3_O_4_@C core–shell NP-based ferrofluids were measured using different concentrations of NPs and with different sized NPs. Exceptional results were obtained, where the electrical conductivity was enhanced up to ∼3222% and ∼2015% for Fe_3_O_4_ (*ca.* 5 nm) and Fe_3_O_4_@C core–shell (*ca.* 7 nm) NP-based ferrofluids compared to the base fluid, respectively. Similarly, an enhancement in the thermal conductivity of ∼153% and ∼116% was recorded for Fe_3_O_4_ (*ca.* 5 nm) and Fe_3_O_4_@C core–shell (*ca.* 7 nm) NPs, respectively. The exceptional enhancement in the thermal conductivity of the bare Fe_3_O_4_ NP-based ferrofluid compared to that of the Fe_3_O_4_@C core–shell NP-based ferrofluid was due to the more pronounced effect of the chain-like network formation/clustering of bare Fe_3_O_4_ NPs in the base fluid. Finally, the experimental thermal conductivity results were compared and validated against the Maxwell effective model. These results were found to be better than results reported till date using either the same or different material systems.

## Abstract 2

Ferrofluids prepared by dispersing superparamagnetic Fe_3_O_4_@C core–shell nanoparticles in water exhibited exceptional enhancement in thermal conductivity without an external magnetic field.

## Graphical abstract

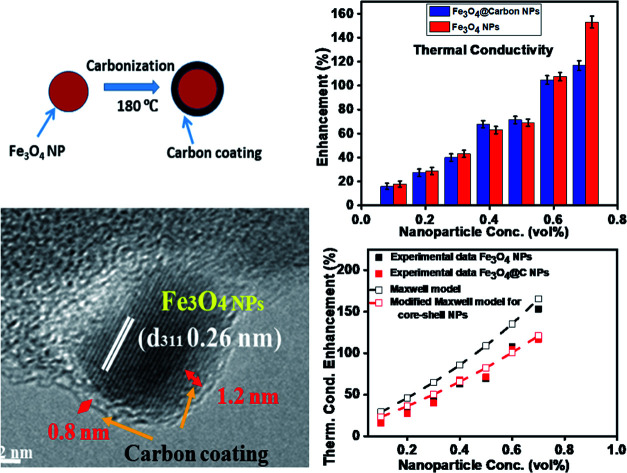

## 1. Introduction

The dispersion of nanoparticles (NPs) in various fluids enhances their thermal conductivity, which have potential application in heat transfer as coolants in various systems such as automobile radiators, refrigerators, process engineering systems, electronic devices, solar energy heaters, pulsating pipes, and thermosyphons.^1–5^ There are several typical base fluids used for heat transfer applications such as water, ethylene glycol, ethanol, methanol, dimethyl formamide, polyalfaolefin, and oils.^6–13^ Ferrofluids or magnetic fluids are stable colloidal homogeneous suspensions of magnetic NPs (∼10 nm in diameter) in an aqueous or a non-aqueous carrier liquid.^14^ The dispersion of NPs in these base fluids results in the formation of a nanofluid, and if NPs are magnetic in nature, the resulting fluid is termed a ferrofluid. Generally, NPs show enhanced electrical, thermal, optical and mechanical properties because of their large surface to volume ratio.^15–17^ Magnetic NPs such as Fe_3_O_4_ have attracted great interest from the scientific community because of their unique properties such as biocompatibility, high electrochemical response and thermal stability.^18^ Consequently, Fe_3_O_4_ NP-based ferrofluids exhibit enhanced thermal and electrical conductivities.^19–21^ However, NPs have high surface energy, which results in their agglomeration or settling at the bottom of the dispersion liquid when used for a long period. Therefore, the coating of inorganic or organic materials over Fe_3_O_4_ NPs is necessary for their good dispersion and long-term stability.^18^ The oxidation of magnetite (Fe_3_O_4_) NPs leads to the formation of a stable phase of iron oxide, which is γ-Fe_2_O_3_ NPs (maghemite), and a more stable form, *i.e.*, α-Fe_2_O_3_ (hematite) under particular conditions.^22^ Therefore, it is necessary to protect superparamagnetic Fe_3_O_4_ NPs from physical and chemical changes by encapsulating them with other materials. Accordingly, capping Fe_3_O_4_ NPs with another sub-material (shell) can result in their good dispersion and stability, as reported by Sharma *et al.*^18^ Hence, various core–shell NPs have been reported thus far, especially Fe_3_O_4_@C core–shell NPs, for *e.g.*, Xuan *et al.* synthesized carbon-encapsulated Fe_3_O_4_ core–shell particles *via* the reduction of glucose.^23^ Conversely, Wang *et al.* synthesized single carbon layer-coated ultra-small Fe_3_O_4_ NPs using a one-step hydrothermal technique for surface-enhanced Raman spectroscopy studies,^24^ and He *et al.* used *in situ*-synthesized carbon-encapsulated Fe_3_O_4_ NPs as an anode material for lithium ion batteries.^25^ Moreover, Zhao *et al.* employed the hydrothermal method for the synthesis of interconnected carbon nanospheres covering Fe_3_O_4_ NPs.^26^ Similarly, Liang *et al.* synthesized Fe_3_O_4_@Au core–shell NPs for the ultrasensitive detection of carbohydrate–protein interactions.^27^ A magnetic metal nanocomposite was coated by carbon to prepare FeNi@C core–shell NPs.^28^ Li *et al.* synthesized Cu@C core–shell NPs *via* a simple state reduction method and investigated their optical properties.^29^ Similarly, Ag@C core–shell NPs were synthesized *via* the wet chemical route and catalysed under hydrothermal conditions, which exhibited hydrophilic and unique optical properties.^30^ Moreover, the reverse micelle method was employed to prepare Fe@Fe-oxide and Fe@Au core–shell nanostructures.^31,32^ These core–shell NPs possessed an added advantage over other NPs as their shell can protect their core from physical and chemical changes. Thus, bio-incompatible, highly reactive and toxic NPs can be covered with biologically compatible, non-reactive and environmentally friendly materials in the form of shells.

To further explore the unique properties of core–shell NPs, they were introduced in a dispersion liquid to make stable ferrofluids for various applications. Recently, ferrofluids were prepared by dispersing Fe_3_O_4_ NPs in mineral oil and γ-Fe_2_O_3_ NPs in various base fluids.^19–21^ Their thermal conductivities were enhanced multiple folds compared to that of the base liquids. Similarly, Cui *et al.* synthesized superparamagnetic Fe_3_O_4_ NPs, which were dispersed in perfluoropolyether base fluid, and the resulting ferrofluid exhibited enhanced viscosity and thermal conductivity.^33^ Zupan *et al.* prepared a ferrofluid by dispersing iron(ii,iii) oxide NPs in water for heat transfer application.^34^ Furthermore, Fe_3_O_4_ NPs were dispersed in water and a maximum 200% enhancement in thermal conductivity was observed.^35^

Core–shell NP-based ferrofluids also exhibit good biocompatibility and improved properties. Accordingly, the other applications of core–shell NP-based ferrofluids include multimodal imaging, hyperthermia, drug delivery, cytocompatibility tumour targeting, cancer chemotherapy and thermotherapy and other possible biological applications.^36–38^ Similarly, magnetic NP systems are widely used in the MRI-guided delivery of magneto-electric drug nano-carriers to the brain.^39^ Magneto-electric core–shell NPs exhibit enhanced cell uptake and control drug release under the influence of an applied and magnetic field.^40^ To manage central nervous system (CNS) diseases, surface-engineered magnetic NPs have been employed as a tool *via* image-guided therapy and theranostics.^41^ Magneto-electric NPs (MENPs) are stimulus-responsive nanosystems for controlled drug release and cell uptake.^42,43^ The futuristic application of these magnetic core–shell nanostructures may be projected in the biomedical field, where previous studies showed the use of magnetic core–shell nanoparticles as an antimicrobial agent.^44^ Targeted drug delivery and drug delivery to the brain are limited due to their complicated methods and structural behavior. Thus, to achieve this goal, magnetic core–shells have been suggested as important nanocarriers.^45,46^

Although nanofluids based on non-magnetic NPs are widely studied, to date, few studies investigating the thermal conductivity of magnetic core–shell NPs for heat transfer applications have been performed.^47–49^ To the best of our knowledge, the electrical and thermal conductivities of superparamagnetic Fe_3_O_4_@C core–shell NPs have rarely been reported. Therefore, herein, we synthesized Fe_3_O_4_ NPs using a chemical co-precipitation method followed by the hydrothermal synthesis technique, which led to the formation of Fe_3_O_4_@C core–shell NPs. Furthermore, these NPs were characterized using various techniques and their electrical and thermal conductivities were measured. The super-paramagnetic Fe_3_O_4_@C core–shell NPs exhibited good thermal and electrical properties, and thus can be exploited for heat transfer applications, such as cooling of electronic devices, fuel cells, and solar cells.^50^

## 2. Experimental

### 2.1. Materials and methods

Ferrous sulphate heptahydrate (FeSO_4_·7H_2_O), ferric chloride hexahydrate (FeCl_3_·6H_2_O) and sodium hydroxide (NaOH) were purchased from Sigma Aldrich. Absolute alcohol and hydrochloric (HCl) acid were procured from Chem-Lab (Belgium) and Scharlau (Spain), respectively. All chemicals were of research grade and used without further purification. Similarly, fructose of analytical grade was purchased from Gem-Chem (India) and used as received. Deionized (DI) water obtained from an ultrapure water unit (Puris-Expe water system) was used during the synthesis of the Fe_3_O_4_ NPs.

### 2.2. Synthesis of Fe_3_O_4_ NPs

Fe_3_O_4_ NPs were synthesized *via* a chemical co-precipitation method. In a typical co-precipitation method, 0.32 mol of ferrous sulphate heptahydrate (FeSO_4_·7H_2_O) and 0.64 mol of ferric chloride were dissolved in 100 mL DI water separately. The Fe^2+^/Fe^3+^ ions were mixed properly before dispersing them in an alkali solution. The ion mixture was then transferred to a 100 mL burette. In a 250 mL round-bottom flask, 1.5 M NaOH solution was prepared and the volume was made up to 100 mL. The iron salt ions were then added dropwise to the above alkali solution under vigorous stirring. The pH of the solution was maintained at around 11–12 during the reaction. An additional amount of NaOH solution could be added to the reaction vessel if needed. As soon as the iron salts were added to the alkali solution, precipitation of the salts started, resulting in the formation of a black-colored co-precipitate. The precipitate was then stirred for 30 min at room temperature. Subsequently, the solution was allowed to settle for 30 min and with the help of a strong magnet, the precipitate was separated from the unreacted solution. Finally, the obtained precipitate was washed several times with distilled water. Similarly, by tuning the pouring of aqueous solution of iron salt (drops) into the aqueous solution of alkali and stirring rate, Fe_3_O_4_ NPs with three different sizes were obtained including 5 nm, 12 nm and 25 nm.

### 2.3. Synthesis of Fe_3_O_4_@C core–shell NPs

The as-synthesized Fe_3_O_4_ NPs of different sizes were dispersed separately in a 5 M aqueous solution of glucose and kept in a tight capped 500 mL bottle, which was half-filled with the solution. To coat a carbon shell over the Fe_3_O_4_ NPs, the sample solution was shaken properly and then left in an autoclave for 4–5 h at high pressure and 180 °C. The carbonization of glucose occurred at 180 °C during the hydrothermal treatment. After this reaction, the system was allowed to cool to room temperature. A dark black-colored solution smelling like sugarcane vinegar was obtained, which was decanted with the help of a strong magnet. Subsequently, the sample was washed with distilled water several times. Finally, the black-colored precipitate was collected and dried at 80 °C for 4–5 h in an oven.^18,23^ Consequently, Fe_3_O_4_@C core–shell NPs with different sizes including 7 nm, 14 nm and 28 nm were obtained using this technique.

### 2.4. Preparation of ferrofluids

We introduced fine powders of Fe_3_O_4_ and Fe_3_O_4_@C core–shell NPs into DI water in separate beakers. Given that the NPs were highly magnetic in nature, the mixtures were sonicated for 3–4 hours for better dispersion of the NPs. Eventually, we obtained a highly monodispersed NP solution. The prepared ferrofluids were found to be stable without any external applied magnetic field for a long time. Before their application in a heat exchanger, the ferrofluids were sonicated again for better heat transfer results. Different concentrations of ferrofluids were prepared in a similar way for heat transfer and electrical conductivity measurements.

### 2.5. Characterization

The phase and crystallinity of the as-synthesized Fe_3_O_4_ and Fe_3_O_4_@C powder samples were identified by XRD (D8 AαS Advance X-ray diffractometer using Cu Kα radiation, *λ* = 1.54156 Å). Raman analyses were conducted using a Raman spectrometer (Aramis LabRam spectrometer). The microstructure analyses of the as-synthesized powders were carried out using a transmission electron microscope (TEM) (JEOL, JEM2100F) operated at 200 kV. X-ray photoelectron spectroscopy (XPS) was performed for elemental information using an X-ray photoelectron spectrometer (Thermo Fisher, USA). Moreover, the functional groups were identified using FT-IR (ATR-FT-IR Nicolet iS 10). The magnetic properties of the as-synthesized powders were analyzed using an ADE 3473-70 Technologies vibrating sample magnetometer (VSM) in the range of −10 kOe to 10 kOe with a magnetization error of ±1%. The zeta potential values of the prepared ferrofluids were obtained using a Zetasizer (Nano-ZS; Malvern, UK) operated at room temperature. The electrical conductivities of the ferrofluids were measured using an HI 2300 NaCl/TDS/EC meter (HANNA Instruments). For the measurement of the thermal conductivity of the ferrofluids with different concentrations, a WL-373 (GUNT Hamburg, Germany) instrument was used. This unit is particularly suited for the determination of the coefficients of thermal conduction of liquids and gaseous materials. The unit was comprised of a double-walled cylinder with an integrated heater acting as the heat source, and the surrounding cylinder as the heat sink. There was a narrow slot in the unit, which was sufficient to prevent heat by convection, and therefore the heat of transfer in the slot is due to the thermal conduction. Due to the constant width of the slot, thermal conduction occurs in a plane wall. The heat transferred, *Q*, can be calculated using Fourier's law as follows:1
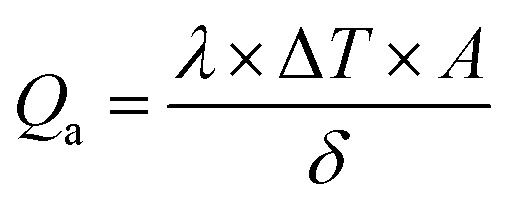
where *Q*_a_ = heat transfer, *λ* = thermal conductivity coefficient, Δ*T* = temperature gradient, *A* = surface area and *δ* = thickness of the slot.

## 3. Results and discussion

**Fig. 1:**
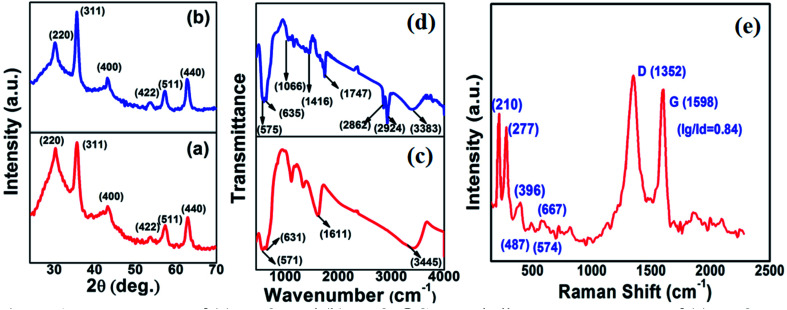
XRD patterns of (a) Fe_3_O_4_ and (b) Fe_3_O_4_@C core–shell NPs. FTIR spectra of (c) Fe_3_O_4_ and (d) Fe_3_O_4_@C core–shell NPs and (e) Raman spectrum of Fe_3_O_4_@C core–shell NPs.

Fig. 1a and b show the XRD patterns of the as-synthesized Fe_3_O_4_ powder and Fe_3_O_4_@C powder samples, respectively. Fig. 1a shows diffraction peaks at 2*θ* values of around 30.09°, 35.45°, 43.02°, 53.59°, 57.20° and 62.72°, which correspond to the (220), (311), (400), (422), (511) and (440) crystal planes, respectively. The peak positions and relative intensities of the Fe_3_O_4_ powder sample matched with the JCPDS card no. 19-629, which confirmed the crystalline nature and cubic spinel structure of the powder sample. Similarly, for the Fe_3_O_4_@C powder sample, the diffraction peaks at around 30.2°, 35.5°, 43.17°, 53.59°, 57.37° and 62.72° correspond to the (220), (311), (400), (422), (511) and (440) crystal planes, respectively, as shown in Fig. 1b.^18,23^ There is a negligible shift in the diffraction peaks, which suggest that the Fe_3_O_4_ powder sample did not transform into another iron oxide phase during the synthesis of the Fe_3_O_4_@C powder sample.^18,23^Fig. 1c and d show the FT-IR spectra of the Fe_3_O_4_ and Fe_3_O_4_@C powder samples, respectively. The peaks observed at 571 and 631 cm^−1^ correspond to the vibrations of Fe–O (Fig. 1c).^18,23,51^ However, for the Fe_3_O_4_@C powder sample, the Fe–O vibration bands were observed at 575 and 635 cm^−1^. The small shift in the Fe–O vibration band is due to the carbon coating over Fe_3_O_4_. Moreover, the decrease in the vibration frequency of the bond indicates the addition of carbon. The peaks at 2924 cm^−1^ and 2862 cm^−1^ refer to the C–H stretching vibrations, and the peaks at 1416 cm^−1^ and 1066 cm^−1^ refer to the C–H bending vibrations and C–O stretching vibrations, respectively, for the Fe_3_O_4_@C NPs. The broad band observed at 3300–3450 cm^−1^ and the band at 1611 cm^−1^ present in both spectra correspond to the O–H stretching mode and the H–O–H bending mode, respectively, indicating the presence of interstitial water molecules.^18,23,52^ The Raman spectrum of the Fe_3_O_4_@C powder sample is shown in Fig. 1e, where the intense D band and G band were observed at around 1352 and 1598 cm^−1^, respectively. The D band can be attributed to sp^2^ carbon, which indicates a disordered graphitic structure and the G band can be attributed to sp^3^ carbon. The other significant peaks at around 396 cm^−1^ and 487 cm^−1^ in the Raman spectrum suggest the T_2g_ phonon vibration mode in Fe_3_O_4_.^53^ The peaks at 574 cm^−1^ and 667 cm^−1^ suggest the Fe=O vibration, which is known as the A_1g_ phonon vibration mode and generally observed due to the symmetric stretching of the O atom along the Fe–O bonds in Fe_3_O_4_.^53^

**Fig. 2:**
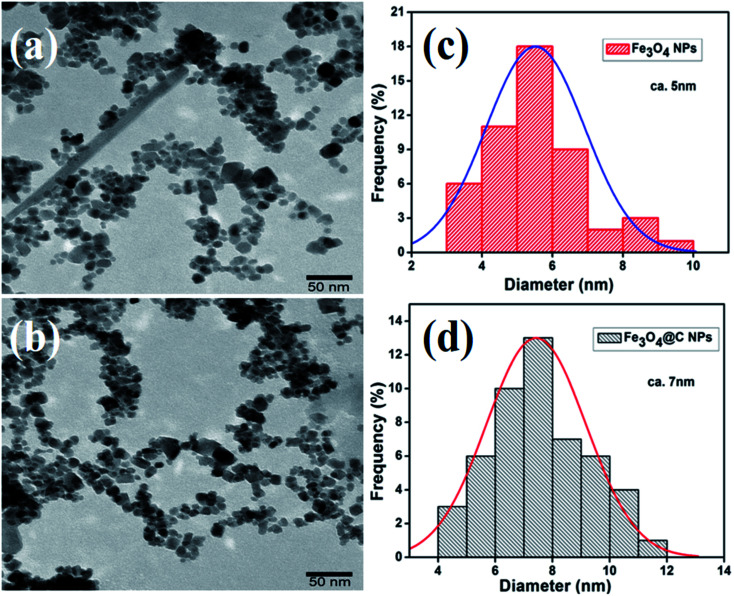
Low magnification TEM images of (a) Fe_3_O_4_ NPs (5 nm) and (b) Fe_3_O_4_@C core–shell NPs (7 nm) and (c) and (d) their corresponding size distribution histograms, respectively.

**Fig. 3:**
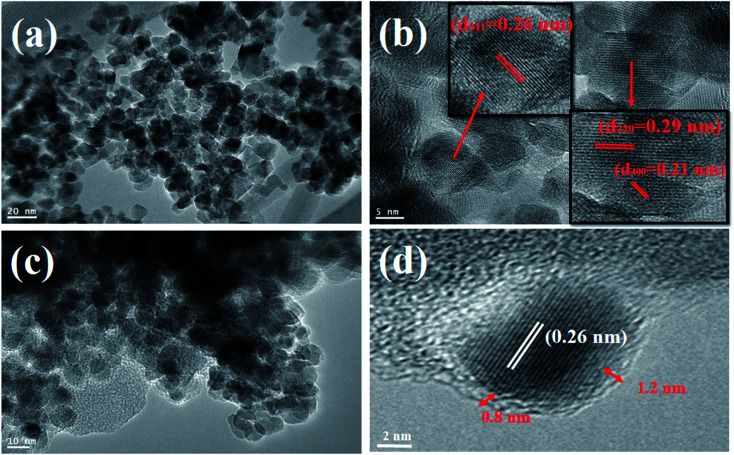
High-magnification TEM images of (a) Fe_3_O_4_ NPs and (c) Fe_3_O_4_@C core–shell NPs. (b) and (d) HRTEM images of Fe_3_O_4_ NPs and a random Fe_3_O_4_@C core–shell NP.

Fig. 2a and b show the low-magnification TEM images of the as-synthesized Fe_3_O_4_ and Fe_3_O_4_@C powder samples, and their corresponding size distribution histograms are shown in Fig. 2c and d, respectively. It was observed that the as-synthesized powder samples were comprised of NPs, which were monodispersed on the TEM Cu grid. The average particle size of the Fe_3_O_4_ NPs and Fe_3_O_4_@C NPs were found to be around 5 nm and 7 nm, respectively. Similarly, the TEM images of two different sized Fe_3_O_4_ NPs (12 nm and 25 nm) and Fe_3_O_4_@C core–shell NPs (14 nm and 28 nm) together with their corresponding size distribution histograms are shown in Fig. S1 (ESI†). Fig. 3a and b show the high-magnification TEM and HRTEM images of the Fe_3_O_4_ NPs, respectively. As shown in Fig. 3b, three different types of planes were identified with the *d* spacings of 0.29, 0.26 and 0.21 nm, which correspond to the (220), (311) and (400) planes of cubic Fe_3_O_4_.^53^ The HRTEM results also confirmed the crystalline nature of the as-synthesized Fe_3_O_4_ NPs and suggest that they did not transform into another stable form of iron oxide. Similarly, Fig. 3c and d show the high-magnification TEM and HRTEM images of the carbon-encapsulated Fe_3_O_4_ (Fe_3_O_4_@C) NPs, respectively. The carbon-shell coating over the Fe_3_O_4_ NPs was found to be amorphous in nature, and the thickness of the carbon shell over a random Fe_3_O_4_ core NP was found to be approximately in the range of 0.8–1.2 nm.

**Fig. 4:**
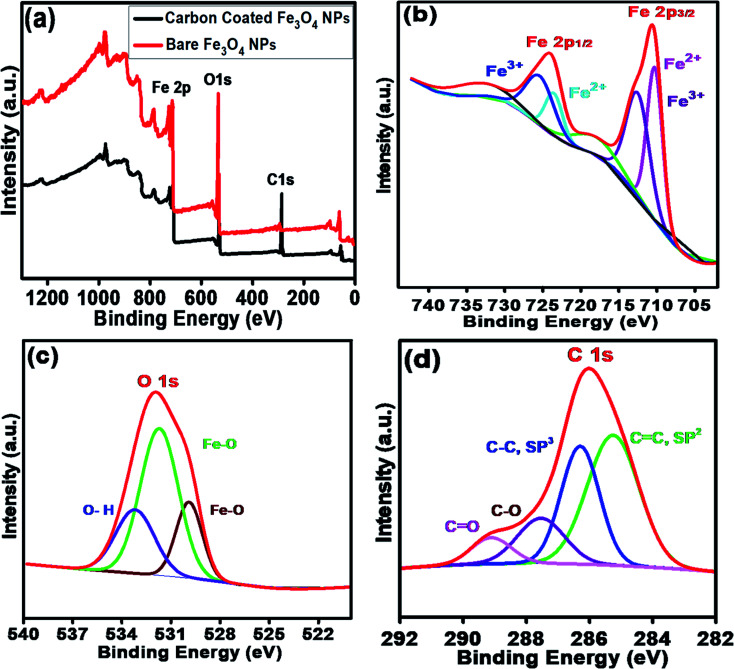
(a) XPS survey scan of bare Fe_3_O_4_ NPs and carbon-coated Fe_3_O_4_ (Fe_3_O_4_@C) core–shell NPs. HR scans of (b) Fe 2p_3/2_ and Fe 2p_1/2_ and (c) O 1s and (d) C 1s core level spectra.

Fig. 4a shows the XPS survey scans of the bare Fe_3_O_4_ NPs (red color) and Fe_3_O_4_@C core–shell NPs (black color). Three significant peaks corresponding to the Fe 2p, O 1s and C 1s core levels were obtained for the carbon-coated Fe_3_O_4_ NPs, which confirmed the composition of the Fe_3_O_4_@C core–shell NPs, whereas the C 1s core level peak was missing for the bare Fe_3_O_4_ NPs. Fig. 4b shows the high-resolution spectrum of Fe 2p core level, *i.e.*, Fe 2p_3/2_ and Fe 2p_1/2_, which correspond to the binding energies 710.5 eV and 724.1 eV, respectively. No peak was observed at 719 eV, which corresponds to α-Fe_2_O_3_, indicating that no phase transformation occurred.^53^ Fe exists in two states, *i.e.*, Fe^2+^ and Fe^3+^, in Fe_3_O_4_. The peak at 710.3 eV (pink) is attributed to the Fe^2+^ state in Fe 2p_3/2_. The peak observed at 712.7 eV (purple) corresponds to the Fe^3+^ state in Fe 2p_3/2_. Similarly, for Fe 2p_1/2_, a peak appeared at 725.7 eV (blue), which correlates to Fe^3+^, and another peak appeared at 723.7 eV (cyan), corresponding to Fe^2+^. The HR O 1s core level spectrum is shown Fig. 4c. The peaks at 531.7 eV (green) and 529.9 eV (wine) are attributed to the Fe–O bond in the Fe_3_O_4_ NPs. The other peak observed at 533.1 eV (blue) corresponds to the O–H bond. The HR C 1s core level spectrum is shown in Fig. 4d. The peaks at 28.63 eV (blue) and 285.2 eV (green) correspond to the C–C sp^3^ and C=C sp^2^ carbon, respectively. The small peaks observed at 287.5 eV (purple) and 289.1 eV (pink) are attributed to C–O and C=O, respectively, which may originate from airborne organic contaminants. Moreover, it was also confirmed that no reaction between the Fe_3_O_4_ NPs and carbon coating occurred.

**Fig. 5:**
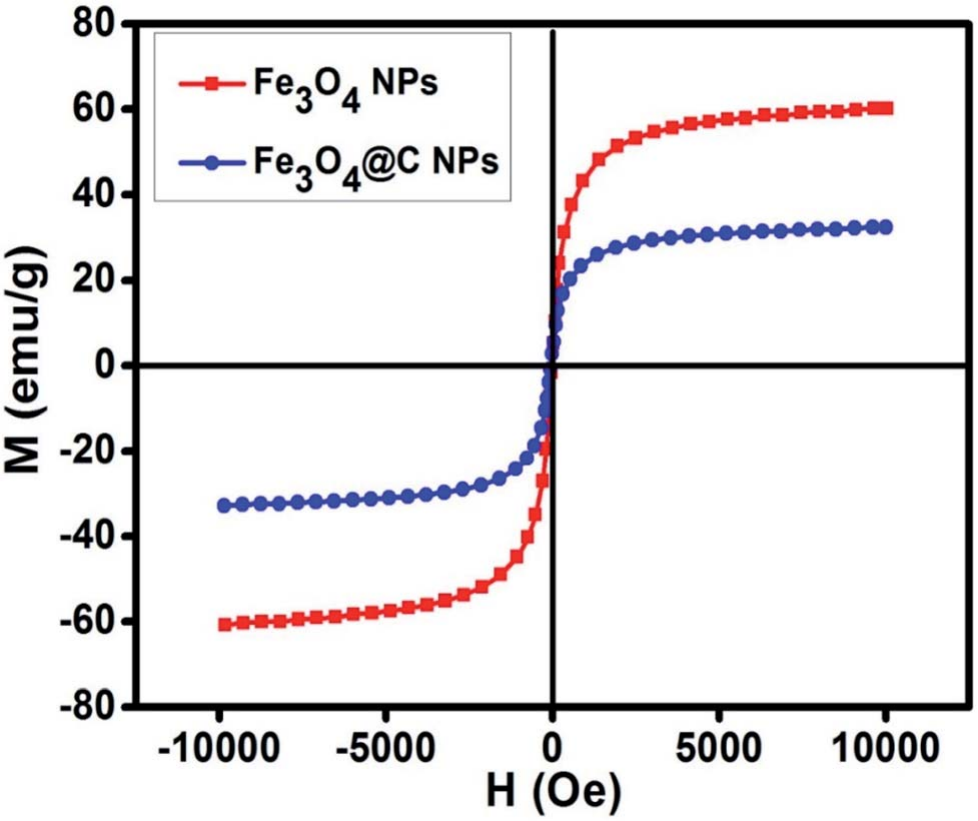
Magnetic hysteresis loop of Fe_3_O_4_ NPs (*ca.* 5 nm) and Fe_3_O_4_@C core–shell NPs (*ca.* 7 nm).

**Fig. 6:**
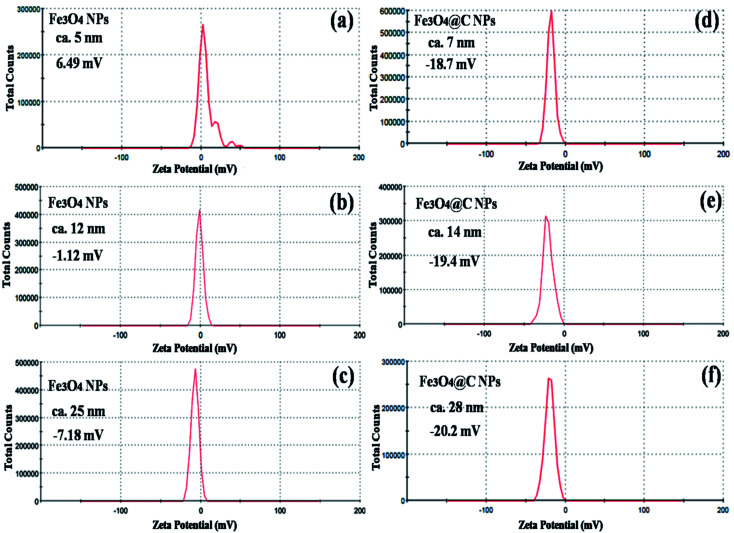
Zeta potential plots of (a) 5 nm Fe_3_O_4_, (b) 12 nm Fe_3_O_4_, (c) 25 nm Fe_3_O_4_, (d) 7 nm Fe_3_O_4_@C, (e) 14 nm Fe_3_O_4_@C and (f) 28 nm Fe_3_O_4_@C NP-based ferrofluids at 0.7 vol% NP concentration.

Fig. 5 shows the magnetic characterization of the small average-sized Fe_3_O_4_ NPs (*ca.* 5 nm) and Fe_3_O_4_@C core–shell NPs (*ca.* 7 nm) with respect to different magnetic fields at 300 K. An unusual behavior in magnetic properties was observed in NPs compared to their bulk counterpart. The magnetization was observed to be 60 emu g^−1^ for Fe_3_O_4_ NPs (*ca.* 5 nm), and it was obvious that there was no coercivity and remanence. Hence, the as-synthesized Fe_3_O_4_ NPs (*ca.* 5 nm) were found to be superparamagnetic in nature.^24^ Similarly, the as-synthesized Fe_3_O_4_@C core–shell NPs (*ca.* 7 nm) also exhibited super-paramagnetic behavior, and the magnetization was found to be 30 emu g^−1^ with no coercivity and remanence. The saturation magnetization was found to be much lower in the case of the core–shell NPs. The low value of the magnetic saturation may be attributed to the carbon coating over the Fe_3_O_4_ NPs as carbon materials are diamagnetic in nature.^54^ A low coercive field was also observed in the case of both NPs which indicate their spherical shape.^55^ The stabilities of the prepared ferrofluids were evaluated in terms of zeta potential and the results are shown in Table 1 and Fig. 6. The zeta potentials were measured for the different sized Fe_3_O_4_ and Fe_3_O_4_@C NP-based ferrofluids at 0.7 vol% of NPs. The results suggest coagulation/agglomeration behavior for the Fe_3_O_4_ NP-based ferrofluids (zeta potential <±5), leading to the formation of networks, whereas the Fe_3_O_4_@C NP-based ferrofluids showed moderate stability (±10 < zeta potential < ±30).^56,57^ An increase in the stability of NPs or their good dispersibility in the base fluid is generally observed for core–shell NPs.^58,59^

**Table 1:** Zeta potential of the different sized Fe_3_O_4_ and Fe_3_O_4_@C NP-based ferrofluids at 0.7 vol% of NPs

Ferrofluid sample	Nanoparticles	Size (nm)	Zeta potential (mV)
1	Fe_3_O_4_	5	6.49
2	Fe_3_O_4_	12	−1.12
3	Fe_3_O_4_	25	−7.18
4	Fe_3_O_4_@C	7	−18.7
5	Fe_3_O_4_@C	14	−19.4
6	Fe_3_O_4_@C	28	−20.2

**Fig. 7:**
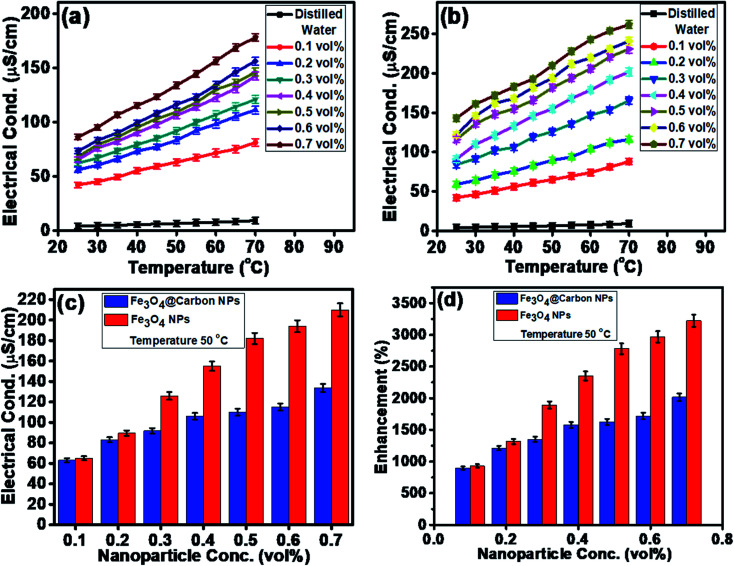
Electrical conductivities of (a) Fe_3_O_4_@C NP (7 nm)-based ferrofluids and (b) Fe_3_O_4_ NP (5 nm)-based ferrofluids at different temperatures. (c) Comparison of the electrical conductivities of the Fe_3_O_4_ and Fe_3_O_4_@C core–shell NP-based ferrofluids at 50 °C and (d) Comparison of the percentage enhancement in electrical conductivity for the Fe_3_O_4_ and Fe_3_O_4_@C core–shell NP-based ferrofluids at 50 °C with respect to DI water.

The electrical conductivities (ECs) of the as-synthesized Fe_3_O_4_ NPs (*ca.* 5 nm) and Fe_3_O_4_@C core–shell NP (*ca.* 7 nm)-based ferrofluids with different concentrations and temperature were measured. The EC of the ferrofluid was found to increase with an increase in the concentration of both types of NPs, as shown in Fig. 7a and b. Fig. 7a shows the ECs of distilled water (base fluid) and the ferrofluids with different concentrations of Fe_3_O_4_@C core–shell NPs at various temperatures. The EC of the ferrofluids was found to be many times higher than that of the base fluid. Similarly, Fig. 7b shows the ECs of Fe_3_O_4_ NP-based ferrofluids, which followed the same trend as observed in Fig. 7a for the Fe_3_O_4_@C core–shell NP-based ferrofluids. However, the ECs of the bare Fe_3_O_4_ NP-based ferrofluids were found to be higher than the core–shell NP-based ferrofluids. This may be attributed to the amorphous nature of the carbon coating, which resulted in a decrease in the EC. Fig. 7c shows a comparison of the ECs of the Fe_3_O_4_ and Fe_3_O_4_@C core–shell NP-based ferrofluids at 50 °C. It was quite obvious that the Fe_3_O_4_ NP-based ferrofluid at a particular concentration of Fe_3_O_4_ NPs exhibited a higher EC compared to the core–shell NP-based ferrofluid. Fig. 7d demonstrates the percentage enhancement in the EC compared to that of the base fluid. It was found that the Fe_3_O_4_ NP-based ferrofluid exhibited the highest value of EC, *i.e.*, 205 μS cm^−1^ for 0.7 vol% of NPs at 50 °C. Conversely, the EC value of 130 μS cm^−1^ was recorded for the Fe_3_O_4_@C core–shell NP-based ferrofluid at the same concentration and temperature. The percentage enhancement was calculated using the following equation:2
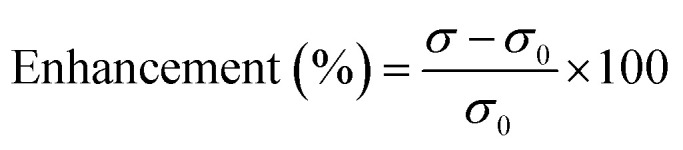
where *σ*_0_ = EC of DIW and *σ* = EC of the ferrofluid.

The maximum enhancement in the EC of the Fe_3_O_4_ NP- and Fe_3_O_4_@C core–shell NP-based ferrofluids was obtained as ∼3222% and ∼2015%, respectively, for 0.7 vol% of NPs at 50 °C. There are many factors that affect the EC of a nanofluid or a ferrofluid such as volume fraction, temperature, structure of NPs, types of NPs, and types of fluids.^60^ The EC of a nanofluid/ferrofluid generally increases with an increase in the volume fraction of the nanofluid/ferrofluid and with temperature. The ions on the surface of the colloidal NPs are attracted to the oppositely charged ions, which results in the formation of a layer. Another layer of ions is also formed, where the ions from the suspension become attached to the first layer by Coulomb force, which electrically screens the first layer. The ions in the second layer are loosely associated and move due to the electric force of attraction and thermal motion. Hence, as the temperature increases, the free ions move rapidly into the suspension of the ferrofluid, which results in a high EC. An increase in the concentration of NPs also enhanced the EC as more solid NPs suspended into the fluid were available to expose their surfaces toward the ions and formed an electrical double layer. However, a relatively low EC in the case of the Fe_3_O_4_@C core–shell NPs was observed as the carbon-coated suspended Fe_3_O_4_ NPs had a lower number of electrons on their surfaces to get attracted toward the free ions compared to the bare Fe_3_O_4_ NPs. The electrons were more populated near the Fermi level in the semiconductor (Fe_3_O_4_) NPs compared to the non-metallic amorphous carbon. Hence, relatively low ECs were observed for the Fe_3_O_4_@C core–shell NP-based ferrofluids compared to the Fe_3_O_4_ NP-based ferrofluids.

**Fig. 8:**
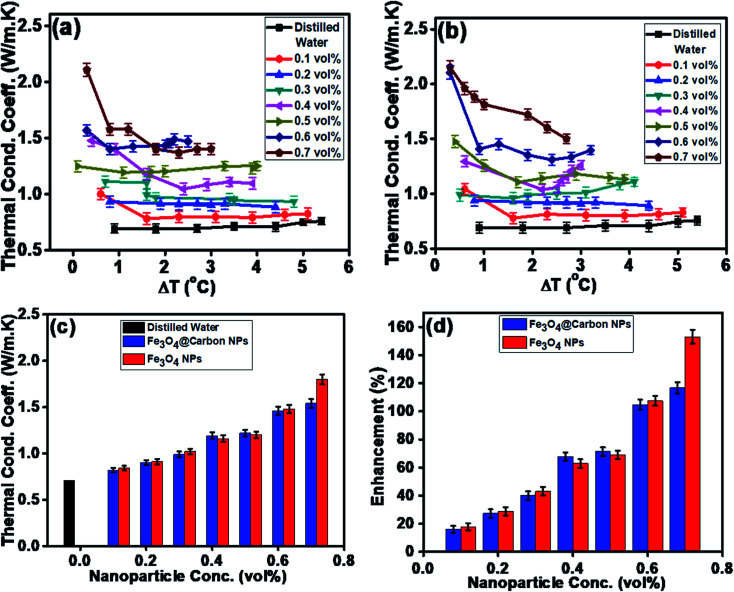
Thermal conductivity with Δ*T* (a) Fe_3_O_4_@C core–shell NP (7 nm)-based ferrofluids and (b) Fe_3_O_4_ NP (5 nm)-based ferrofluids. (c) Comparative thermal conductivity coefficient and (d) comparative percent enhancement in thermal conductivity of Fe_3_O_4_ NP- and Fe_3_O_4_@C core–shell NP-based ferrofluids with respect to DI water.

Similarly, the thermal conductivities of the Fe_3_O_4_ NP- and Fe_3_O_4_@C core–shell NP-based ferrofluids were measured. Fig. 8a and b show the graphs of the coefficient of thermal conductivity *versus* temperature gradient for the Fe_3_O_4_@C core–shell NP (7 nm)- and Fe_3_O_4_ NP (5 nm)-based ferrofluids, respectively. For both types of NPs, with concentrations in the range of 0.1 vol% to 0.7 vol%, it was observed that the coefficient of thermal conductivity (*λ*) increased consistently. The average thermal conductivity coefficients for water was found to be 0.71 W m^−1^ K^−1^, and for the ferrofluids at different concentrations of Fe_3_O_4_@C core–shell NPs, *i.e.*, 0.1, 0.2, 0.3, 0.4, 0.5, 0.6 and 0.7 vol% they were found to be 0.82, 0.90, 0.99, 1.19, 1.22, 1.46 and 1.54 W m^−1^ K^−1^, respectively, as shown in Fig. 8a. A similar trend was observed for the Fe_3_O_4_ NP-based ferrofluids, and the values of the coefficient of thermal conductivity for various concentrations, *i.e.*, 0.1, 0.2, 0.3, 0.4, 0.5, 0.6 and 0.7 vol% were obtained as 0.84, 0.92, 1.02, 1.16, 1.21, 1.48 and 1.80 W m^−1^ K^−1^, respectively, as shown in Fig. 8b. The comparison of thermal conductivities and percentage enhancement of Fe_3_O_4_ NP- and Fe_3_O_4_@C core–shell NP-based ferrofluids are shown in Fig. 8c and d, respectively. Fig. 8d depicts the increase in percentage enhancement with respect to DI water from 16% to 116% as the concentration of Fe_3_O_4_@C core–shell NPs varied from 0.1 vol% to 0.7 vol%. Similarly, a 17% to 153% enhancement with respect to DI water was observed for the Fe_3_O_4_ NPs as their concentration varied from 0.1 vol% to 0.7 vol%.

**Fig. 9:**
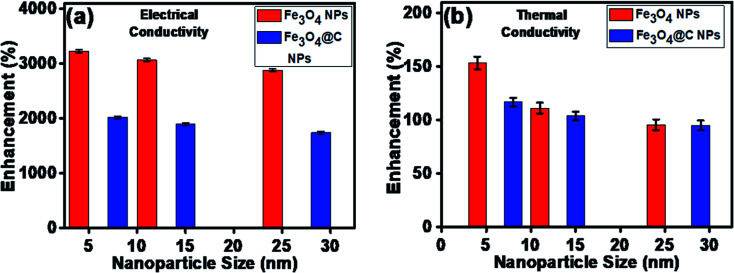
Effect of NP size on (a) electrical conductivity enhancement and (b) thermal conductivity enhancement of ferrofluids.

The maximum enhancement was observed for 0.7 vol% of Fe_3_O_4_ NPs. This may be attributed to the clustering effect of the NPs as their surface energies were quite high owing to their high surface to volume ratio and the formation of a chain-like network of small-sized NPs, which increased the effective volume fraction of heat conductive phases in the ferrofluid. Moreover, the dispersed NPs in the water-based ferrofluid executed Brownian motion, which led to collisions between the NPs and with the molecules of the water.^61,62^ Conversely, in the case of the Fe_3_O_4_@C core–shell NPs, the thermal capacity of the carbon coating and percolation pathways for heat conduction were responsible for the thermal conductivity enhancement.^61,62^ It should be noted that at only 0.7 vol%, the thermal conductivity of the Fe_3_O_4_@C core–shell NP-based ferrofluid was significantly less than that of the Fe_3_O_4_ NP-based ferrofluid. Moreover, the effects of different NPs sizes on the enhancement of the electrical and thermal conductivities were also studied, as shown in Fig. 9a and b (Fig. S2 and S3, ESI†), respectively. Fig. 9a shows the variation in the electrical conductivity enhancement of the Fe_3_O_4_ NP- and Fe_3_O_4_@C NP-based ferrofluids with NP size. The EC was found to be enhanced with a decrease in the size of the NPs for both types of ferrofluids. The increase in EC with a decrease in the NP size is attributed to the increase in surface area and electrophoretic mobility of the NPs.^63,64^ A similar trend was observed for the thermal conductivity enhancement, which increased with a decrease in NP size for both types of ferrofluids, as shown in Fig. 9b. The exceptional enhancement in the thermal conductivity especially in the case of the bare Fe_3_O_4_ NP (5 nm)-based ferrofluid compared to the larger-size Fe_3_O_4_ NP (12 nm and 25 nm)-based ferrofluids is due to the more pronounced effect of chain-like network formation/clustering of the NPs, as inferred from the zeta potential values (Table 1 and Fig. 6). However, owing to the carbon coating over the bare Fe_3_O_4_ NPs, relatively better dispersion and less agglomeration of the different sized Fe_3_O_4_@C NPs (as inferred from their more negative zeta potential values, Table 1 and Fig. 6) in the base fluid (water) resulted in a lower enhancement in thermal conductivity for the Fe_3_O_4_@C NP-based ferrofluids compared to the Fe_3_O_4_ NP-based ferrofluids.

**Fig. 10:**
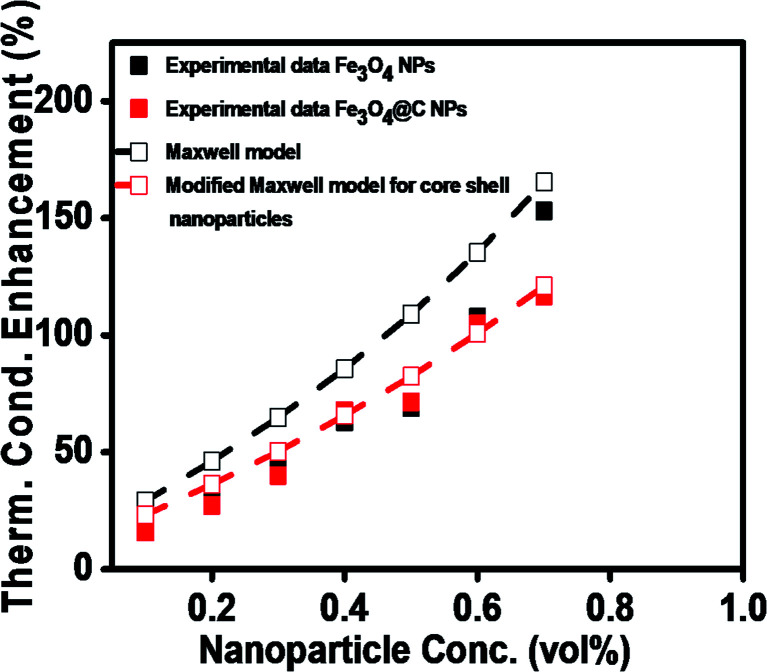
Comparison of experimental and predicted values of thermal conductivities of Fe_3_O_4_ (*ca.* 5 nm) and Fe_3_O_4_@C (*ca.* 7 nm) NP-based ferrofluids.

To theoretically validate the obtained results, the experimental values of the thermal conductivities of the Fe_3_O_4_ NP (*ca.* 5 nm)- and Fe_3_O_4_@C NP (*ca.* 7 nm)-based ferrofluids were fitted with the existing Maxwell model,^65^ as shown in Fig. 10. Based on the effective medium theory, the effective thermal conductivity of the Fe_3_O_4_ NPs coated with carbon was calculated using the following equation:^66^3
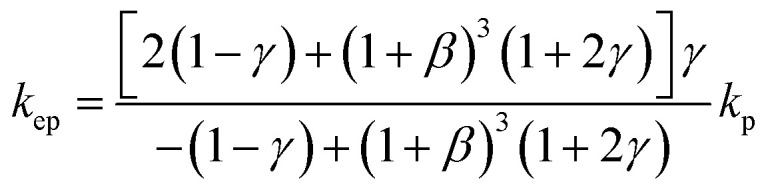
where *k*_ep_ is the equivalent thermal conductivity of the Fe_3_O_4_@C NPs, *k*_p_ is the thermal conductivity of the Fe_3_O_4_ NPs, *γ* is the ratio of coated nanolayer thermal conductivity to the particle thermal conductivity, and *β* is the ratio of coated layer thickness to that of the particle radius. The thermal conductivity of the ferrofluids based on Fe_3_O_4_ NPs and Fe_3_O_4_@C NPs was calculated using the Maxwell equation as follows:4
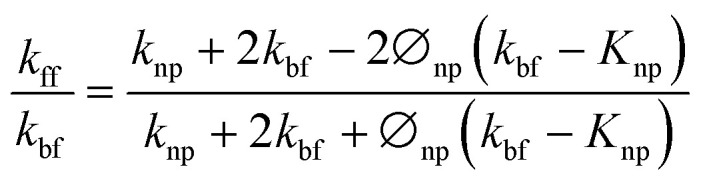
where *k*_ff_, *k*_bf_ and *k*_np_ are thermal conductivities of the ferrofluid, base fluid and NP (*k*_ep_ was used instead of *k*_np_ in the case of the carbon-coated Fe_3_O_4_ NPs), respectively, and ⌀_np_ is the concentration of NPs (vol%) in the base fluid. It can be seen in Fig. 10 that the values predicted by the Maxwell model are higher at low concentrations for the Fe_3_O_4_ NP (*ca.* 5 nm)-based ferrofluids. In contrast, for the Fe_3_O_4_@C (*ca.* 7 nm) NP-based ferrofluid, the predicted values of thermal conductivity closely fit the experimental data. The slight deviation in the thermal conductivity values of the Fe_3_O_4_ NP-based ferrofluids is attributed to the clustering or aggregation of the NPs, as inferred from the zeta potential values (Table 1 and Fig. 6).^67^ Therefore, the Maxwell model validated the experimental results for the Fe_3_O_4_@C NP-based ferrofluid. These obtained results were compared with the results reported in the literature, as presented in Table 2.

**Table 2:** Comparison of the electrical and thermal conductivities of our Fe_3_O_4_ and Fe_3_O_4_@C core–shell NP-based ferrofluids with that in the literature

Material	Size (nm)	Conc./volume fraction	Base fluid	Electrical conductivity enhancement (%)	Thermal conductivity enhancement (%)	Ref.
Fe_3_O_4_	14.2	0.5	Water	360	—	68
SiO_2_	12	1.1–2.4	Water	—	1.1	69
TiO_2_	27	3.1–4.3			10.8	
Al_2_O_3_	13	1.3–4.3			32.4	
Graphene	—	0.5/0.1	Ethylene glycol/water	1664	10.47	70
Fe@C	—	1.5 wt%	Polyethylene glycol	—	30	39
Al@C					40	
Cu@C					49	
Iron oxide(ii,iii)	50	1 g L^−1^	Water	—	34	34
Fe_3_O_4_	5–10	2.5 g L^−1^	Mineral oil	—	51	19
γ-Fe_2_O_3_	12	1.5 g L^−1^	50 : 50 oil mixture	—	51	21
CuO	29	0–6	Ethylene glycol/water	—	60	71
Al_2_O_3_	53	0–10			69	
γ-Fe_2_O_3_	13	1.5 g L^−1^	Liquid paraffin/sunflower/mineral oil	—	20.6	20
					45.2	
					77	
Fe_3_O_4_@C	7	0.7 vol%	Water	2015	116	This work
Fe_3_O_4_ NPs	5			3222	153	

## 4. Conclusion

In conclusion, Fe_3_O_4_ and Fe_3_O_4_@C core–shell NP-based ferrofluids were prepared to investigate their thermal and electrical conductivities. Firstly, we synthesized different sized Fe_3_O_4_ NPs *via* a chemical co-precipitation method using iron salts and alkali solution as precursors. The successfully synthesized Fe_3_O_4_ NPs were introduced in an autoclave with a solution of glucose at 180 °C and high pressure. The hydrothermal process enabled the formation of a carbon shell over the Fe_3_O_4_ NPs. The average particle sizes of the Fe_3_O_4_ and Fe_3_O_4_@C core shell NPs were found to be in the range of 5–25 nm and 7–28 nm, respectively. The structural and elemental analyses revealed that no phase transformation of Fe_3_O_4_ occurred during the formation of core–shell NPs. The magnetic characterization revealed that the as-synthesized small average-sized Fe_3_O_4_ NPs (*ca.* 5 nm) and Fe_3_O_4_@C core–shell NPs (*ca.* 7 nm) were superparamagnetic in nature. Finally, the electrical and thermal conductivities of the Fe_3_O_4_ and Fe_3_O_4_@C core–shell NP-based ferrofluids were measured at different concentrations of NPs and with different sized NPs. Exceptional results were obtained, where the electrical conductivity was enhanced up to ∼3222% and ∼2015% for the Fe_3_O_4_ (*ca.* 5 nm) and Fe_3_O_4_@C (*ca.* 7 nm) core–shell NP-based ferrofluids, respectively, compared to distilled water (base fluid). Similarly, the enhancement in the thermal conductivity was determined to be ∼153% and ∼116% for the Fe_3_O_4_ (*ca.* 5 nm) and Fe_3_O_4_@C (*ca.* 7 nm) core–shell NPs, respectively.

## Author contributions

Mohd Imran: Conceptualization, methodology, investigation, writing-original draft preparation, supervision; Nasser Zouli: visualization, investigation, supervision; Tansir Ahamad: characterization, investigation, funding; Saad M. Alshehri: characterization, investigation, funding; Mohammed Rehaan Chandan: visualization, validation, review & editing; Shahir Hussain: conceptualization, methodology, investigation, electrical conductivity experiment; Abdul Aziz: resources, thermal conductivity experiment; Mushtaq Ahmad Dar: data curation, characterization; Afzal Khan: visualization investigation, supervision, review & editing.

## Conflicts of interest

The authors declare no competing financial interest.

## Supplementary Material

NA-003-D1NA00061F-s001
