# Supplementary material for: Carbon-coated Fe3O4 core–shell super-paramagnetic nanoparticle-based ferrofluid for heat transfer applications
Source: Nanoscale Adv. 2021 Feb 8;3(7):1962–75. doi: 10.1039/d1na00061f (PMC9418956; doi:10.1039/d1na00061f)
Supplement: NA-003-D1NA00061F-s001 [file NA-003-D1NA00061F-s001.pdf]

Electronic Supplementary Material (ESI) for Nanoscale Advances.
This journal is © The Royal Society of Chemistry 2021

Supplementary Information

Carbon Coated Fe3O4 Core-shell Super-paramagnetic Nanoparticles Based Ferrofluid for Heat Transfer Application

Mohd Imran[1], Nasser Zouli[1], Tansir Ahamad[2], Saad M Alshehri[2], Mohammed Rehaan Chandan[3], Shahir Hussain[4], Abdulaziz[5], Mushtaq Ahmad Dar[6], Afzal Khan[7]*

[1]*Department of Chemical Engineering, Faculty of Engineering, Jazan University, P.O. Box. 706, Jazan 45142, Saudi Arabia*

[2]*Department of Chemistry, College of Science, King Saud University, P.O. Box 2455, Riyadh 11451, Saudi Arabia*

[3]*Colloids and Polymers Research Group, School of Chemical Engineering, Vellore Institute of Technology, Vellore, Tamilnadu 632014, India*

[4]*Department of Electrical Engineering, Faculty of Engineering, Jazan University, P.O. Box. 706, Jazan 45142, Saudi Arabia*

[5]*Department of Mechanical Engineering, Faculty of Engineering, Jazan University, P.O. Box. 706, Jazan 45142, Saudi Arabia*

[6]*Center of Excellence for Research in Engineering Materials, College of Engineering, King Saud University, Riyadh, 11421, Kingdom of Saudi Arabia*

[7]*State Key Laboratory of Silicon Materials, School of Materials Science and Engineering, Zhejiang University, Hangzhou-310027, China*

TEM images and Size Distribution

TEM analyses were carried out for the prepared different size of Fe_3O_4 and Fe_3O_4@C core-shell nanoparticles. TEM images were selected for the size analyses with the help of ImageJ software by considering around 50 nanoparticles for the average size distribution. Size distribution histograms were also plotted for the respective TEM images and presented in Fig.S1. The average sizes of Fe_3O_4 nanoparticles were measured to be 12 nm and 25 nm. The TEM images show the agglomeration in Fe_3O_4 nanoparticles due to high surface energy [1]. Similarly, average sizes of 14 nm and 28 nm were obtained for Fe_3O_4@C core-shell nanoparticles.

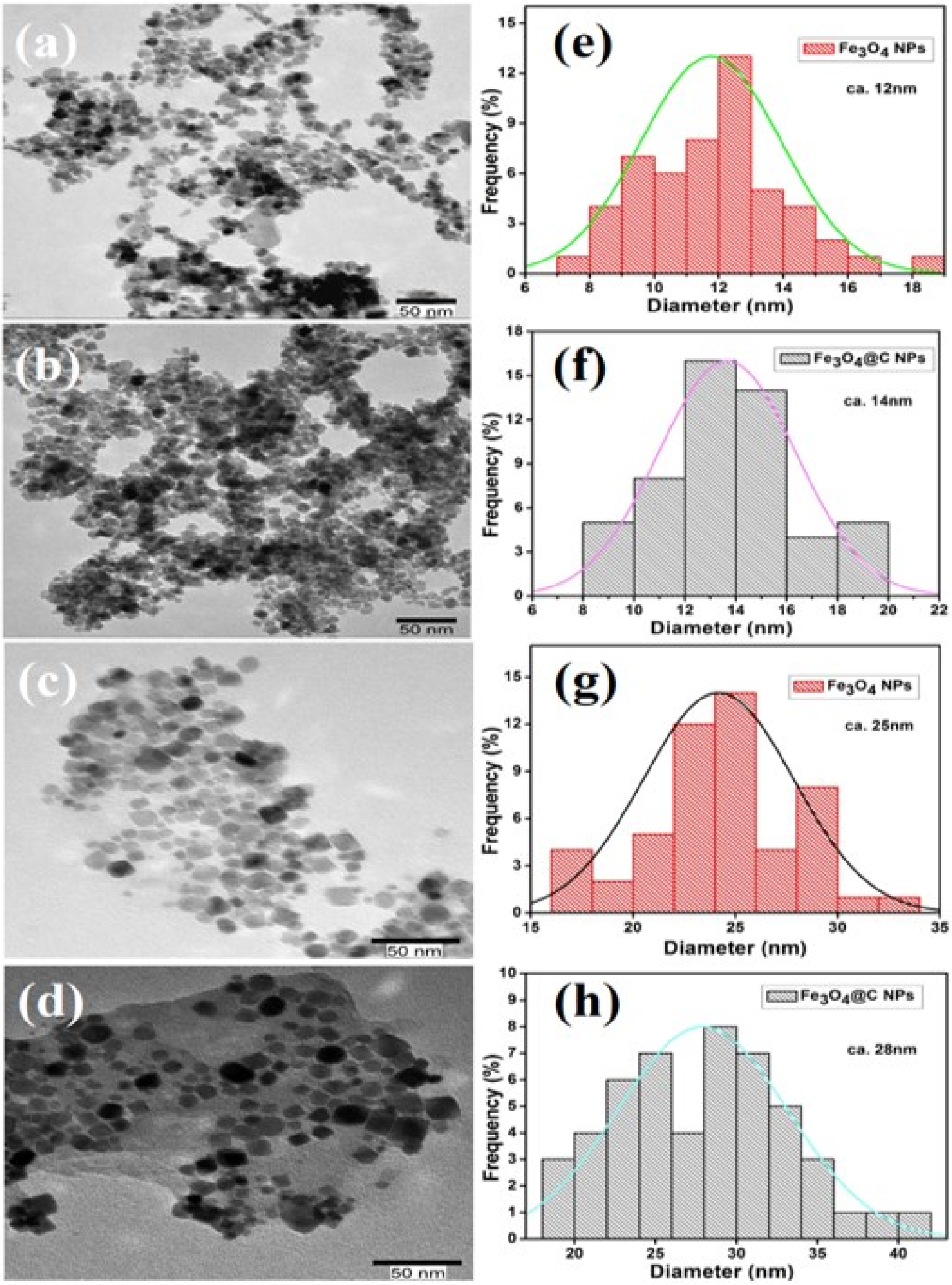

Figure S1: TEM images of Fe_3O_4 nanoparticles (a, c) and Fe_3O_4@C core-shell nanoparticles (b, d) and Size distribution histogram of Fe_3O_4 nanoparticles (e, g) and Fe_3O_4@C core-shell nanoparticles (f, h).

Effect of Nanoparticles Size on Electrical and Thermal Conductivities of Ferrofluids:

(a) Electrical Conductivity

The electrical conductivities (ECs) of ferrofluids based Fe_3O_4 NPs of 12 nm and 25 nm were measured with different concentrations and temperature. The EC of the ferrofluid was found to be increased with an increase in the concentration and temperature [3]. The ECs of the ferrofluids was found to be many times higher than the EC of the base fluid (De-ionize water). Similar trends were followed by the Fe_3O_4@C core-shell NPs based ferrofluids. However, the ECs of bare Fe_3O_4 NPs based ferrofluids were found to be higher than the core-shell NPs based ferrofluids. **Fig.S2a** demonstrates the percent enhancement in the EC for 12 nm sized Fe_3O_4 NPs based ferrofluid. It was found that the Fe_3O_4 NPs based ferrofluid exhibited the maximum enhancement i.e. 3064.5% for 0.7 Vol.% of NPs at 50 °C. In the same way, percent enhancement in EC of 25 nm size Fe_3O_4 NPs based ferrofluid were found to be 2874.6 % for 0.7 Vol.% of NPs as shown in **Fig.S2c**. On the other hand, the EC was recorded for Fe_3O_4@C core-shell NPs based ferrofluid at the same concentration and temperature. The percent enhancement in EC of 14 nm and 28 nm sized Fe_3O_4@C core-shell NPs based ferrofluid were found to be 1893.6 % and 1735.4% respectively for 0.7 Vol.% of NPs as shown in **Fig.S2 (b, d)**. It is observed that (i) the Fe_3O_4 NPs based ferrofluids have higher electrical conductivity than Fe_3O_4@C core-shell NPs based ferrofluids under the same conditions and (ii) electrical conductivity of smaller sized NPs found to be higher than the large sized NPs [4].

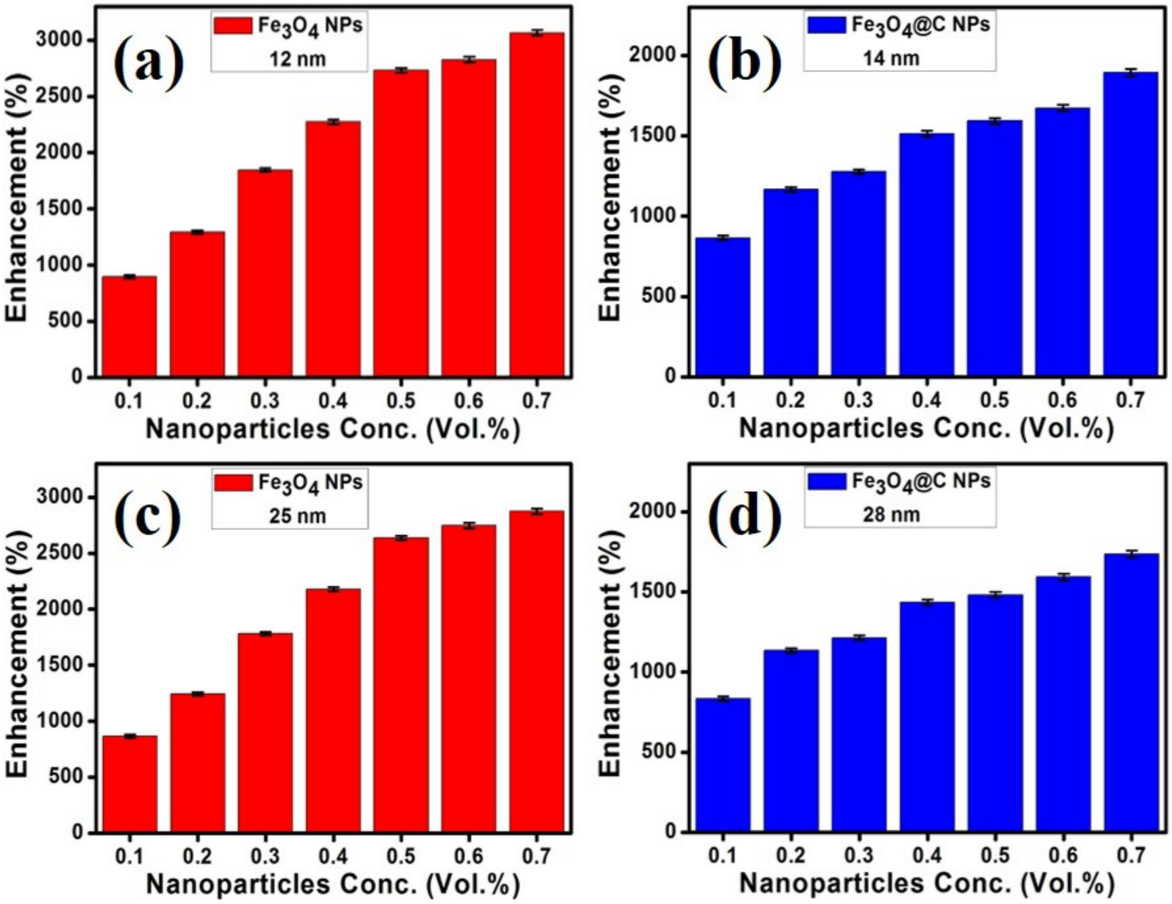

Figure S2: Percent enhancement in electrical conductivity (a, c) Fe_3O_4 nanoparticles and (b, d) Fe_3O_4@C core-shell nanoparticles based ferrofluids on varying concentration of nanoparticles at 50 °C temperature.

(b) **Thermal Conductivity**

The thermal conductivity coefficient of larger size magnetite NPs (12 nm and 25 nm) and core-shell NPs (14 nm and 28 nm) were measured in the same way as it was measured for 5 nm (Fe_3O_4) and 7 nm (Fe_3O_4@C core-shell NPs) (as presented in the main manuscript). The thermal conductivity coefficient of water and ferrofluids were calculated by Fourier's law. Then, % enhancement in thermal conductivity of ferrofluids calculated against the thermal conductivity of base fluid (water), presented in **Fig.S3**. The thermal conductivity of ferrofluids increased with an increase in vol.% of the NPs [5]. **Fig.S3** (a) presents the percent enhancement of 12 nm sized Fe_3O_4 NPs. The highest thermal conductivity enhancement i.e. ~111 % was obtained at 0.7 vol.

% of NPs. Similarly, for 25 nm sized Fe_3O_4 NPs, it is found to be 95.4% at 0.7 vol.% of NPs as shown in **Fig.S3b**. Furthermore, the thermal conductivity enhancement was calculated as ~104 % and ~95 % for Fe_3O_4@C core-shell NPs of 14 nm and 28 nm size, respectively, at 0.7 vol. % of NPs. Thus, it is noted that Fe_3O_4 and Fe_3O_4@C core-shell NPs based ferrofluids exhibits higher thermal conductivity for smaller sized NPs as compared to larger sized NPs. The increase in thermal conductivity with decrease in particles size is in agreement with the previously published research [6, 7].

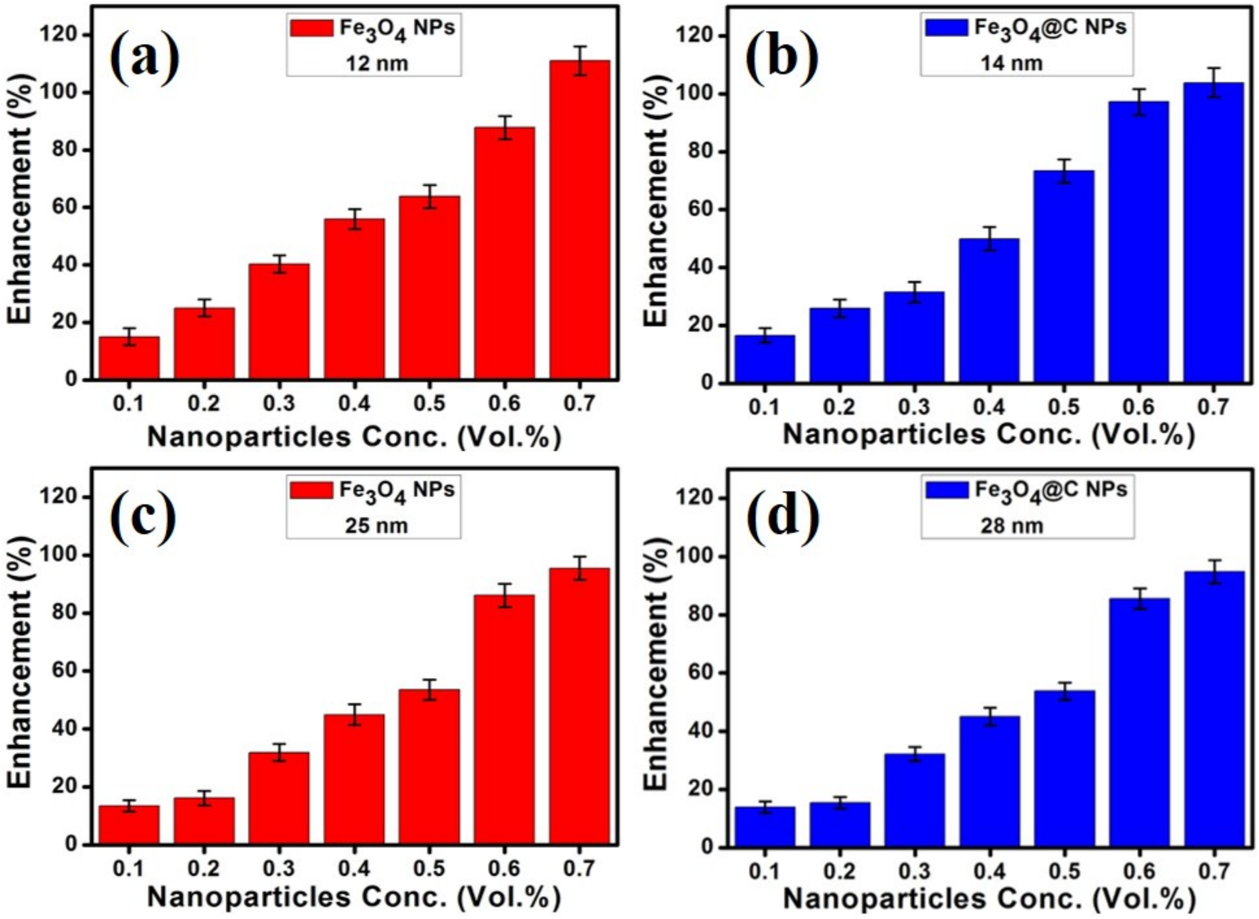

Figure S3: Percent enhancement in thermal conductivity of (a, c) Fe_3O_4 nanoparticles (12 nm and 25 nm) and (b, d) Fe_3O_4@C core-shell nanoparticles (14 nm and 28 nm) based ferrofluids at varying concentration of nanoparticles.

References:

1. Chakraborty, S.; Sharma, K. S.; Rajeswari, A.; Vimalnath, K. V.; Sarma, H. D. Pandey, U.; et al. Radiolanthanide-loaded agglomerated Fe_3O_4 nanoparticles for possible use in the treatment of arthritis: formulation, characterization and evaluation in rats. *J. Mater. Chem. B* 3, **2015,**27, 5455-5466.
2. Fal, J.; Wanic, M.; Malicka, M.; Oleksy, M.; Żyła. G. Experimental investigation of electrical conductivity of ethylene glycol containing indium oxide nanoparticles. *Actaphys. Polon. A* **2019**,135, 1237-1239.
3. Sarojini, K. G. K.; Manoj, S. V.; Singh, P. K.; Pradeep, T.; Das. S. K.; Electrical conductivity of ceramic and metallic nanofluids. *Colloids Surf. A: Physicochem. Engg. Asp.* **2013**, 417 39-46.
4. Hussain, S.; Alam, M. M.; Imran, M.; Zouli, N.; Aziz, A. Irshad, K.; et al. Fe_3O_4 nanoparticles decorated multi-walled carbon nanotubes based magnetic nanofluid for heat transfer application. *Mater. Lett.* **2020,** 274,128043.
5. Iyahraja, S.; Rajadurai. J. S.; Study of thermal conductivity enhancement of aqueous suspensions containing silver nanoparticles. *AIP Adv.* 5, **2015**, 5, 057103.
6. Warrier, P.; Teja, A.; Effect of particle size on the thermal conductivity of nanofluids containing metallic nanoparticles. *Nanoscale Res. Lett.* **2011**, 6, 1, 1-6.
